# The association between sarcopenia and incident of depressive symptoms: a prospective cohort study

**DOI:** 10.1186/s12877-023-04653-z

**Published:** 2024-01-18

**Authors:** Zhenzhen Li, Bingqing Liu, Xiang Tong, Yao Ma, Ting Bao, Jirong Yue, Chenkai Wu

**Affiliations:** 1 Health Management Center, General Practice Center, West China Hospital of Sichuan University, Chengdu, China; 2 Biostatistics Group, Data Management and Statistics Department, Haisco Pharmaceutical Group, Chengdu, China; 3 Department of Respiratory and Critical Care Medicine, West China Hospital of Sichuan University, Chengdu, China; 4 Department of Geriatrics and National Clinical Research Center for Geriatrics, West China Hospital, Sichuan University, Guoxuexiang 37, Chengdu, Sichuan 610041 China; 5 Department of Public Health, School of Health Sciences and Practice, New York Medical College, Valhalla, NY USA; 6 School of Biological and Population Health Sciences, Oregon State University, Corvallis, OR USA

**Keywords:** Sarcopenia, Low muscle mass, Low muscle strength, Depression, Depressive symptoms

## Abstract

**Background:**

Epidemiological studies have shown that sarcopenia was associated with depression among older adults. However, most of these investigations used a cross-sectional design, limiting the ability to establish a causal relation, the present study examined whether sarcopenia was associated with incident depressive symptoms.

**Methods:**

This is a prospective cohort study with participants from the Western China Health and Aging Trends (WCHAT) study. Participants could complete anthropometric measurements and questionnaires were included. The exposure was sarcopenia, defined according to the Asian Working Group for Sarcopenia in 2019, the outcome was depressive symptoms, evaluated by GDS-15. We excluded depression and depressive symptoms at baseline and calculated the risk of incident depressive symptoms during the follow-up year.

**Results:**

A total of 2612 participants (mean age of 62.14 ± 8.08 years) were included, of which 493 with sarcopenia. 78 (15.82%) participants with sarcopenia had onset depressive symptoms within the next year. After multivariable adjustment, sarcopenia increased the risk of depressive symptoms (RR = 1.651, 95%CI = 1.087–2.507, *P* = 0.0187) in overall participants. Such relationship still exists in gender and sarcopenia severity subgroups. Low muscle mass increased the risk of depressive symptoms (RR = 1.600, 95%CI = 1.150–2.228, *P* = 0.0053), but low muscle strength had no effect (RR = 1.250, 95%CI = 0.946–1.653, *P* = 0.117).

**Conclusions:**

Sarcopenia is an independent risk factor for depressive symptoms, Precautions to early detect and targeted intervene for sarcopenia should continue to be employed in adult with sarcopenia to achieve early prevention for depression and reduce the incidence of adverse clinical outcomes.

**Supplementary Information:**

The online version contains supplementary material available at 10.1186/s12877-023-04653-z.

## Introduction

Sarcopenia is an age-related disease characterized by progressive loss of skeletal muscle mass, muscle strength, and/or physical function. Sarcopenia is prevalent among older adults and is associated with a wide variety of adverse outcomes, such as frailty, falls, disability, and mortality [1]. Recent studies have found that sarcopenia is also related to depression [2]. Aging, physical inactivity and dysregulated levels of inflammatory cytokines or hormones in sarcopenia may be risk factors for depression [3, 4]. Depression is one of the most important causes of emotional distress in later life, significantly reducing quality of life and becoming one of the major diseases contributing to the global disease burden [5].

The latest meta-analysis identified a higher prevalence of depression among sarcopenia patients than in the general population and revealed a significant association between sarcopenia and depression [6]. However, all included studies were cross-sectional in design and could not establish a causal relationship between sarcopenia and depression, and the degree of sarcopenia was not graded in almost all studies, so the effect of varying severity of sarcopenia on the risk of depression is unclear.

Therefore, we examined the association between sarcopenia and incident depressive symptoms among older adults without preexisting depressive symptoms in western China. Furthermore, we examined the association between sarcopenia and incident depressive symptoms among subgroups stratified by baseline age, gender, sarcopenia severity and sarcopenia diagnostic components. This prospective cohort study can confirm the effect of sarcopenia on the risk of depression and provide a theoretical basis for sarcopenia prognosis research and depression risk factor screening in the future.

## Methods

### Ethics

The study was reviewed and approved by the Ethical Committee (approval: 2017 − 445) and all subjects signed informed consent forms, and the cohort study was conducted in accordance with the Declaration of Helsinki.

### Study setting and population

This was a one-year prospective cohort study, the baseline survey was done from July to December 2018 and follow-up information was obtained from July to December 2019 [7].

Participants were from the Western China Health and Aging Trends (WCHAT) study [7]. Participants with 50 years and older and could completed a body composition test, handgrip strength test, 4-meter walking test, and questionnaires were included into the cohort [7]. Participants who had baseline depression and depressive symptoms as assessed by the 15-item geriatric depression scale (GDS-15), severe malnutrition, cancer, or > 5% weight change in the last 3 months were excluded.

### Exposure

The primary exposure was sarcopenia, defined as loss of skeletal muscle mass plus loss of muscle strength and/or reduced physical performance according to the Asian Working Group for Sarcopenia in 2019 (AWGS 2019) criteria [8], and defined participants with low muscle mass, low muscle strength and low physical performance as having “severe sarcopenia.” Muscle mass was assessed by bioimpedance analysis (BIA) using a Body Composition Analyzer (Inbody 770, BioSpace, Seoul, Korea) [9]. Low muscle mass was defined as skeletal muscle mass index (SMMI) less than 7.0 kg/m^2^ for men and less than 5.7 kg/m^2^ for women [8]. Muscle strength was assessed by hand strength using a hand-held dynamometer (EH101; Camry, Zhongshan, China). Low muscle strength was defined as a grip strength of less than 28 kg for men and less than 18 kg for women [8]. Physical performance was assessed by usual gait speed over a 4-meter course. Low physical performance was defined as a usual gait speed of less than 1.0 m/s for both sexes [8].

### Outcomes

The primary outcome was depressive symptoms and was assessed by GDS-15, which was a commonly used screening tool for depression among older adults [10]. The GDS-15 has been found to be highly correlated with the original GDS in the Chinese population, and the reliability and validity of this scale have been established [11]. Participants with a score of ≥ 5 were classified as having depressive symptoms [12].

### Covariates

Demographic information included age, sex, marital status (single, married, divorced), educational level (illiteracy, bachelor’s degree or below, and bachelor’s degree or above), occupation (manual labor and other), and whether they lived alone. The height, weight, and body mass index (BMI) of all the participants were recorded. Healthy lifestyle factors included smoking (yes/no), alcohol use (heavy (0.75 g per day), light, and no drinking). Chronic diseases diagnosed by medical institutions, including cancer, heart disease, kidney disease, high blood pressure, diabetes, chronic obstructive pulmonary disease (COPD), chronic liver disease (CLD), chronic kidney disease (CKD), which were reported by participants or their caregivers. Major trauma and acute illness in the last three months were recorded. The Pittsburgh Sleep Quality Index (PSQI) was used to assess the sleep quality in the last month, scores of 16–21 were defined as poor sleep quality [13]. Cognitive status was assessed using the Short Portable Mental Status Questionnaire (SPMSQ), scores of 0–2 were categorized as normal cognitive function; scores of 3–10 were categorized as cognitive function impairment [14]. Physical disability was assessed using the Barthel index activity of daily living (ADL), and ADL disability was defined as having difficulty in ≥ 1 ADL (defecation, urination, grooming, toileting, eating, transferring, moving, dressing, stairs, bathing) [15]. Social support survey was used Social Support Rating Scale (SSRS), which including 10 items across three dimensions: objective support, subjective support and the utilization of support, the total score was the summary of scores for each item, the higher the score was, the better the social support status [16].

### Statistical analyses

Baseline characteristics are presented using means and standard deviations for continuous variables and frequencies and percentages for categorical variables among the entire sample and by baseline sarcopenia status. We used a t test to compare continuous variables and a chi-square test or Fisher’s exact test to compare categorical variables between persons with and without sarcopenia.

We calculated the risk of incident depressive symptoms during the follow-up year among participants with and without sarcopenia and used a chi-square test or Fisher’s exact test to compare categorical variables between the two groups.

Using univariate analysis to find depressive symptoms and its related factors, then a multicollinearity diagnostic was conducted by calculating the values of tolerance and variance inflation factor (VIF) [17]. The values of tolerance > 0.1 and VIF < 10 were used to indicate the absence of multicollinearity among the dependent variables [17].

Subsequently, we used generalized linear regression models to examine the unadjusted and adjusted associations between sarcopenia and incident depressive symptoms. The adjustment variables incorporated those variables that were considered significant in the univariate analysis. Furthermore, we performed subgroups analysis stratified by baseline gender, age (< 60 years and ≥ 60 years), sarcopenia severity (sarcopenia and severe sarcopenia) and sarcopenia diagnostic components (low muscle mass and low muscle strength) using the same methods as above.

The statistical tests were 2-tailed, and *P* < 0.05 was considered to be statistically significant. SAS version 9.4 (SAS Inc., Chicago, Illinois, USA) was used for data management and analysis.

## Results

### Baseline characteristics of participants

**Fig. 1:**
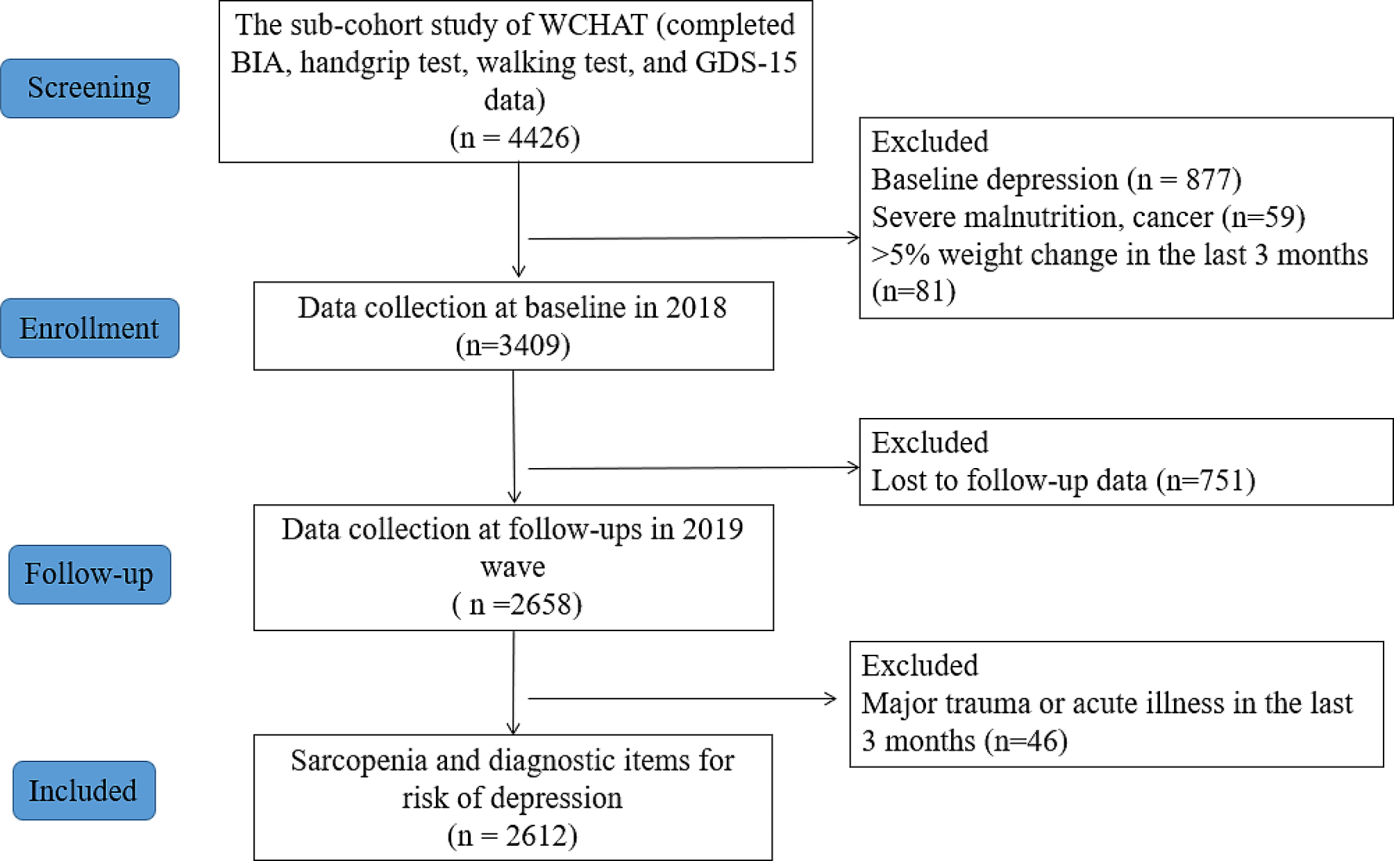
Study flow chart

About 4426 participants completed a body composition test, handgrip strength test, 4-m walking test, and depression assessment. We excluded participants who had baseline depression and depressive symptoms (*n* = 877), severe malnutrition or cancer (*n* = 59), and > 5% weight change in the last 3 months (*n* = 81). A total of 3409 participants were included at baseline in 2018, and we then excluded participants who were lost to follow-up data (*n* = 751) and had major trauma or acute illness in the last 3 months at follow-up assessment (*n* = 46) in 2019 (Fig. 1).

Ultimately, a total of 2612 participants (mean age of 62.14 ± 8.08 years) were analyzed in this study, including 493 patients with sarcopenia and 2119 nonsarcopenia controls. There were differences in age, gender, BMI, skeletal muscle mass, grip strength, walking speed, divorce, widowhood, illiteracy, smoking, cognitive impairment, disability, social support between the two groups, the demographic characteristics are summarized in Table 1.

**Table 1:** Characteristics of the participants at baseline

	Overall (*n* = 2612)	Non-sarcopenia (*n* = 2119)		Sarcopenia (*n* = 493)	P
Age, years	62.14 ± 8.08	60.68 ± 7.55	67.63 ± 8.00		< 0.001
Male, n (%)	1020 (39.05)	786 (37.09)	234 (47.46)		< 0.001
BMI, kg/m2	25.76 ± 4.11	26.56 ± 3.95	22.26 ± 2.84		< 0.001
SMMI, kg/m2	6.73 ± 0.86	6.91 ± 0.79	5.92 ± 0.65		< 0.001
Grip strength, kg	23.54 ± 8.77	24.29 ± 8.84	20.55 ± 7.77		0.026
4-m walking speed, m/s	0.89 ± 0.26	0.91 ± 0.27	0.78 ± 0.20		< 0.001
Divorced, n (%)	44 (1.68)	42 (1.98)	2(0.41)		0.014
Widowed, n (%)	346 (13.25)	258 (12.18)	88 (17.85)		< 0.001
Living alone, n (%)	109 (4.17)	87 (4.11)	22 (4.46)		0.72
Illiteracy, n (%)	623 (23.85)	487 (22.99)	136 (27.59)		0.031
Farming, n (%)	1598 (61.18)	1270 (59.96)	328(66.53)		0.007
Drinking, n (%)	540 (20.67)	427 (20.64)	113 (22.92)		0.2639
Smoking, n (%)	519 (19.87)	378 (17.85)	141 (28.66)		< 0.001
Chronic diseases, n (%)	1216 (46.55)	985(46.51)	231(46.86)		0.8885
Poor sleep quality, n (%)	758 (29.02)	577(27.24)	181(36.71)		0.0589
Cognitive Impairment, n (%)	255 (9.76)	188 (8.87)	67 (13.59)		0.0015
ADL disability n (%)	206 (7.89)	155 (7.31)	51 (10.34)		0.0246
Social support status	43.00 ± 7.07	43.32 ± 6.92	41.59 ± 7.50		< 0.0001

Abbreviations: Values are mean ± SD or valid percentages (n), BMI, body mass index; SMMI, skeletal muscle mass index; ADL, activities of daily living

The sarcopenia group included 424 older people (≥ 60 years old) (Supplementary Table S1), 234 male participants (Supplementary Table S2) and 253 participants with severe sarcopenia (Supplementary Table S3)）. A total of 575 participants with low muscle mass and 980 with low muscle strength in this study (Supplementary Table S4).

Given that 22% of the participants were not followed up after one year, we further compared baseline characteristics with and without follow-up data (Supplementary Table S5). The prevalence of sarcopenia and important confounders did not differ significantly between the two groups.

### Incidence of depression after one-year follow-up

At the one-year follow-up, 78 (15.82%) participants with sarcopenia had onset depressive symptoms. The incidence of depressive symptoms was 10.05% for male participants with sarcopenia, 14.55% for female participants with sarcopenia, 16.75% for older participants with sarcopenia (≥ 60 years old), and 15.42% for participants with severe sarcopenia. 85 (17.78%) participants with low muscle mass and 142 (14.49%) participants with low muscle strength had onset depressive symptoms (Table 2).

**Table 2:** The incident of depressive symptoms in all participants and each subgroup

	Overall		Subgroups for age				Subgroups for gender				Subgroups for sarcopenia severity		Subgroups for sarcopenia diagnostic components			
			< 60 years		≥ 60 years		Male		Female							
	Control (*N* = 2119)	Sarcopenia (*N* = 493)	Control (*N* = 970)	Sarcopenia (*N* = 69)	Control (*N* = 1149)	Sarcopenia (*N* = 424)	Control (*N* = 786)	Sarcopenia (*N* = 234)	Control (*N* = 1333)	Sarcopenia (*N* = 259)	Nonsevere sarcopenia (*N* = 240)	Severe sarcopenia (*N* = 253)	Control 1 (*N* = 2037)	Low muscle mass (*N* = 575)	Control 2 (*N* = 1632)	Low muscle strength (*N* = 980)
Depression, n (%)	273 (12.88)	78(15.82)	139 (14.33)	7 (10.14)	134 (11.66)	71 (16.75)	79 (10.05)	33 (14.1)	194 (14.55)	45 (17.37)	39 (16.25)	39 (15.42)	266(13.06)	85 (17.78)	209 (12.80)	142 (14.49)

### Risk ratios of Sarcopenia to depressive symptoms

In all participants, sarcopenia increased the risk of depressive symptoms (RR = 1.330, 95%CI = 1.041-1.700, *P* = 0.0226) after adjusting for age and gender, and after adjusting for important confounding factors, sarcopenia increased the risk of depressive symptoms (RR = 1.651, 95%CI = 1.087–2.507, *P* = 0.0187) (Table 3).

**Table 3:** Risk ratios of sarcopenia to depressive symptom in overall participants and subgroups

	Model 1		Model 2		Model 3	
	Crude [95% CI]	P	Adjusted RR [95% CI]	P	Adjusted RR [95% CI]	P
**Overall**						
Sarcopenia	1.228 [0.974–1.548]	0.0823	1.330 [1.041-1.700]	0.0226	1.651 [1.087–2.507]	0.0187
**Subgroups for age**						
Age < 60 years	0.708 [0.345–1.453]	0.3464	0.733 [0.357–1.505]	0.3976	1.169 [0.523–2.609]	0.7040
Age ≥ 60 years	1.436 [1.101–1.872]	0.0075	1.500 [1.142–1.971]	0.0036	1.792 [1.219–2.633]	0.0030
**Subgroups for gender**						
Male	1.403 [0.960–2.050]	0.0799	1.476 [0.987–2.207]	0.0580	1.480 [1.028–2.669]	0.0194
Female	1.194 [0.888–1.605]	0.2403	1.253 [0.917–1.712]	0.1574	1.651 [1.087–2.507]	0.0187
**Subgroups for sarcopenia severity**						
Sarcopenia	1.261 [0.927–1.716]	0.1394	1.346 [0.985–1.838]	0.0619	1.520 [1.072–2.376]	0.0464
Severe sarcopenia	1.197 [0.878–1.630]	0.2554	1.313 [0.946–1.823]	0.0103	1.746 [1.108–2.680]	0.0107
**Subgroups for low muscle mass and strength**						
Low muscle mass	1.132 [0.903–1.419]	0.2821	1.213 [0.956–1.539]	0.1122	1.600 [1.150–2.228]	0.0053
Low muscle strength	1.132 [0.928–1.379]	0.2212	1.163 [0.945–1.433]	0.1547	1.250 [0.946–1.653]	0.117

Model 1: No adjustment

Model 2: Adjusted by Age and Gender

Model 3: Adjusted by Age, Gender, BMI, Divorced, Widowed, Living alone, Illiteracy, Social support status, Cognitive Impairment, Sleep quality, ADL disability, Chronic diseases, Smoking, Drinking

After adjusting for important confounding factors, sarcopenia did not increase the risk of depressive symptoms in participants < 60 years old (RR = 1.169, 95%CI = 0.523–2.609, *P* = 0.7040). Sarcopenia increased the risk of depressive symptoms in participants ≥ 60 years old (RR = 1.792, 95%CI = 1.219–2.633, *P* = 0.0030), as well as in male participants (RR = 1.651, 95%CI = 1.087–2.507, *P* = 0.0187) and female participants (RR = 1.792, 95%CI = 1.219–2.633, *P* = 0.0030). In sarcopenia severity subgroup, both nonsevere sarcopenia (RR = 1.520, 95%CI = 1.072–2.376, *P* = 0.0464) and severe sarcopenia (RR = 1.746, 95%CI = 1.108–2.680, *P* = 0.0107) increased the risk of depressive symptoms.

Low muscle mass did not increase the risk of depressive symptoms (RR = 1.213, 95%CI = 0.956–1.539, *P* = 0.1122) after adjusting for age and gender, but after adjusting for important confounding factors, low muscle mass increased the risk of depressive symptoms RR = 1.600, 95%CI = 1.150–2.228, *P* = 0.0053). However, low muscle strength could not increase the risk of depressive symptoms after adjusting for age and gender (RR = 1.163, 95%CI = 0.945–1.433, *P* = 0.1547) or important confounding factors (RR = 1.250, 95%CI = 0.946–1.653, *P* = 0.117).

## Discussion

The results of this study clearly confirmed the causal relationship between sarcopenia and depressive symptoms. Sarcopenia increased the risk of depressive symptoms onset among the older population in western China, as well as in old population and sarcopenia severity subgroups. Compared with muscle strength, low muscle mass was more significant in the risk of depressive symptoms.

The overall prevalence of depression in sarcopenia patients was 0.28 and the overall adjusted odds ratio (OR) between sarcopenia and depression was 1.57 in our recent meta-analysis. In our cohort study, the onset of depressive symptoms in sarcopenia patients was 15.82% overall and 16.71% in patients aged ≥ 60 years, slightly higher than the longitudinal study by Chen et al. [18], which is the only longitudinal study on the incidence of sarcopenia and depressive symptoms to date, showing that the annual incidence of depressive symptoms over the course of the 1-year follow-up was 12.0% for people over the age of 60 years and sarcopenia was significantly associated with depressive symptoms incidence (OR = 3.57, 95% CI = 1.59–8.04). While, in our study sarcopenia was associated with 46.5% higher risks of depressive symptoms (RR = 1.465, 95% CI = 1.141–1.880). These differences are likely due to differences between study populations, varying ethnic characteristics and diagnostic criteria for sarcopenia. In that study, sarcopenia was defined according to the AWGS 2014 criteria and severe sarcopenia was not distinguished.

Studies on the association between muscle mass and muscle strength and depression have been inconsistent. Some studies considered low muscle mass and low muscle strength to be significantly and independently associated with depression, [19] some reported that depression was not associated with low muscle mass but was associated with low muscle strength, [20] and others indicated that depression was significantly correlated with muscle strength but not with muscle mass [21]. However, all the above studies had cross-sectional designs. Fukumori et al. [22] used data from the Locomotive Syndrome and Health Outcomes in the Aizu Cohort Study (LOHAS), and multivariate random-effects logistic analysis revealed that participants with lower grip strength at baseline had higher odds of developing depressive symptoms one year later. Cao’s prospective cohort study including 1,094 participants indicated no significant relationship between baseline grip strength and the risk of developing depressive symptoms during a one-year follow-up period [23]. Our findings are consistent with Chen’s reported that muscle mass rather than muscle strength was associated with new onset depressive symptoms [18]. These differences may be due to the different diagnostic criteria for sarcopenia and depression, differences between the study populations and different ethnic characteristics.

Several mechanisms may be involved in the association between sarcopenia and depression. First, skeletal muscle cells secrete various myokines to regulate their mass and function. Brain-derived neurotrophic factor (BDNF) is a myokine that can cross the blood-brain barrier [24] and is involved in the regulation of neuronal and glial development, neuroprotection, and synaptic interactions [25], several studies have reported decreased serum and plasma BDNF levels in patients with depression [26]. In addition, age-related chronic low-grade inflammation is an important cause of sarcopenia, and low levels of inflammation and oxidative stress also playing a role in depression [27, 28]. Sarcopenia may adversely affect mental function through metabolic and endocrine mechanisms, skeletal muscle is the main organ of glucose homeostasis, and 75% of postprandial glucose intake is completed by skeletal muscle. Low muscle mass may impair glucose homeostasis, and various studies have shown that there is a correlation between blood glucose homeostasis and depression [29]. Disability and insufficient physical activity due to decreased muscle strength and muscle mass may be a cause of depression [30].

This study has several advantages. This was a large, prospective cohort study with rigorous quality control, rigorous assessment of sarcopenia and depressive symptoms, and a complete analysis of sarcopenia diagnostic components on depressive symptoms risk. Despite extensive efforts to curb study limitations, some limitations exist. First, with a follow-up period of only one year, we did not examine the long-term relationship between sarcopenia and depressive symptoms. Second, the attrition rate of follow-up was relatively high, but adults who were lost to follow-up had a similar baseline health status to those who completed follow-up, and incident depressive symptoms should not be affected by missing data. Third, in this study, BIA was used to diagnose low muscle mass, rather than dual energy X-ray absorptiometry (DEXA) or CT, which are inconvenient to perform in large sample surveys in communities and rural areas, and BIA is also recommended as one of the diagnostic methods in AWGS 2019. Finally, our study did not exclude older adults with dementia/cognitive impairment, and GDS assessment results for depressive symptoms may be inadequate for participants with cognitive problems.

## Conclusions

Sarcopenia is an independent risk factor for depressive symptoms, as well as in old population and sarcopenia severity subgroups. Compared with muscle strength, low muscle mass was more significant in the risk of depressive symptoms.

In future clinical studies, precautions to early detect and targeted intervene for sarcopenia should continue to be employed in adult with sarcopenia to achieve early prevention for depression and reduce the incidence of adverse clinical outcomes.

For future research, long-term and large-scale prospective cohort studies with different ages and regions are required to verify the effect of sarcopenia on depression.

### Electronic supplementary material

Below is the link to the electronic supplementary material.

**Supplementary Material 1**: Characteristics of the participants at baseline in subgroups

## Data Availability

The datasets generated and analysed during the current study are not publicly available due the data needs further analysis, but are available from the corresponding author on reasonable request.

## Abbreviations

WCHAT: Western China health and aging trends
RR: Risk ratio
AWGS: Asian working group for sarcopenia
BIA: Bioimpedance analysis
SMMI: Skeletal muscle mass index
GDS: Geriatric depression scale
ADL: Activity of daily living
OR: Odds ratio
DEXA: Dual energy X-ray absorptiometry
