# Supplementary material for: The association between sarcopenia and incident of depressive symptoms: a prospective cohort study
Source: BMC Geriatr. 2024 Jan 18;24:74. doi: 10.1186/s12877-023-04653-z (PMC10797724; doi:10.1186/s12877-023-04653-z)
Supplement: Supplementary file 1 — Supplementary Material 1: Characteristics of the participants at baseline in subgroups [file 12877_2023_4653_MOESM1_ESM.docx]

**Supplementary Material**

Supplementary Table S1. Characteristics of the participants at baseline by age subgroups.

Supplementary Table S2. Characteristics of the participants at baseline by sex subgroups.

Supplementary Table S3. Characteristics of the participants at baseline by sarcopenia severity subgroups.

Supplementary Table S4. Characteristics of the participants at baseline by muscle mass and muscle strength.

Supplementary Table S5. Characteristics of the participants at baseline between follow-up and loss to follow-up.

Supplementary Table S6. Univariate analysis for [depressive symptom](javascript:;).

**Table S1. Characteristics of the participants at baseline by age subgroups.**

|  | <60 years | | | ≥60 years | | |
| --- | --- | --- | --- | --- | --- | --- |
|  | Control  N=970 | Sarcopenia  N=69 | P | Control  N=1149 | Sarcopenia  N=424 | P |
| Age, years | 54.04±2.982 | 55.07±2.58 | 0.002 | 66.63± 4.994 | 69.97 ± 6.60 | <0.001 |
| Male, n (%) | 305 (31.44) | 27 (39.13) | 0.3338 | 481 (41.86) | 207 (48.82) | 0.0136 |
| BMI>30, n (%) | 26.35±4.341 | 22.66±3.667 | <0.001 | 26.74±3.569 | 22.2±2.681 | <0.001 |
| SMMI, kg/m2 | 6.9±0.796 | 5.82±0.627 | <0.001 | 6.93±0.781 | 5.93 ±0.653 | <0.001 |
| Grip strength, kg | 25.53±8.769 | 22.14±8.919 | 0.0031 | 23.24± 8.771 | 20.29 ± 7.54 | <0.001 |
| 4-m walking speed, m/s | 0.94±0.258 | 0.85±0.155 | 0.0051 | 0.89 ± 0.277 | 0.77 ± 0.207 | <0.001 |
| Divorced, n (%) | 21 (2.16) | 0(0) | 0.2169 | 21 (1.83) | 2 (0.47) | 0.0466 |
| Widowed, n (%) | 63 (6.49) | 7 (10.14) | 0.2425 | 195 (16.99) | 81 (19.1) | 0.3273 |
| Living alone, n (%) | 36 (3.71) | 2(2.9) | 0.7282 | 51 (4.44) | 20 (4.72) | 0.8161 |
| Illiteracy, n (%) | 174 (17.94) | 15(21.74) | 0.4291 | 313 (27.26) | 121 (28.54) | 0.6163 |
| Farming, n (%) | 525 (54.12) | 35(50.72) | 0.5842 | 745 (64.9) | 293 (69.1) | 0.1179 |
| Drinking, n (%) | 192 (19.79) | 13(18.84) | 0.8475 | 245 (21.36) | 100 (23.58) | 0.344 |
| Smoking, n (%) | 156 (16.08) | 15(21.74) | 0.2208 | 222 (19.34 | 126 (29.79) | <0.001 |
| Chronic diseases, n (%) | 451 (46.51) | 32(46.3) | 0.6770 | 534 (446.48) | 199 (49.93) | 0.6517 |
| Poor sleep quality, n (%) | 110 (11.34) | 26(37.68) | 0.1321 | 467 (40.64) | 155(36.56) | 0.481 |
| Cognitive Impairment, n (%) | 73 (7.53) | 8(11.59) | 0.2233 | 115 (10.01) | 59 (13.96) | 0.0284 |
| ADL disability, n (%) | 49 (5.05) | 6(8.7) | 0.1915 | 106(9.23) | 45(10.61) | 0.4071 |
| Social support status | 45.02±6.393 | 45.2±6.694 | 0.8232 | 41.89 ±7.029 | 41 ± 7.47 | 0.0326 |

Abbreviations: Values are mean ± SD or valid percentages (n), BMI, body mass index; SMMI, skeletal muscle mass index; ADL, Activities of Daily Living.

**Table S2. Characteristics of the participants at baseline by sex.**

|  | Male | | | Female | | |
| --- | --- | --- | --- | --- | --- | --- |
|  | Control  N=786 | Sarcopenia  N=234 | P | Control  N=1333 | Sarcopenia  N=259 | P |
| Age, years | 62.11±7.763 | 68.19±7.706 | <0.001 | 60.13±7.321 | 67.13±8.243 | <0.001 |
| BMI>30, n (%) | 26.22±3.56 | 21.97±3.115 | <0.001 | 26.77±4.143 | 22.52±2.543 | <0.001 |
| SMMI, kg/m2 | 7.65±0.548 | 6.5±0.365 | <0.001 | 6.48±0.545 | 5.4±0.326 | <0.001 |
| Grip strength, kg | 31.55±8.701 | 25.19±7.997 | <0.001 | 20.03±5.529 | 16.22±4.287 | <0.001 |
| 4-m walking speed, m/s | 0.94±0.259 | 0.79±0.17 | <0.001 | 0.89±0.274 | 0.77±0.229 | <0.001 |
| Divorced, n (%) | 13(1.65) | 1(0.4) | 0.1564 | 29(2.18) | 1(0.39) | 0.0526 |
| Widowed, n (%) | 36 (4.58) | 27 (11.54) | 0.0001 | 222(16.65) | 61(23.55) | 0.0079 |
| Living alone, n (%) | 30(4.07) | 8(3.42) | 0.7753 | 57(4.28) | 14(5.41) | 0.4204 |
| Illiteracy, n (%) | 92(11.7) | 44(18.8) | 0.0052 | 395(29.63) | 92(35.52) | 0.0598 |
| Farming, n (%) | 395(50.25) | 134(57.26) | 0.0619 | 875(65.64) | 197(76.06) | 0.0037 |
| Drinking, n (%) | 300 (28.17) | 88(37.61) | 0.8661 | 137(10.28) | 25(9.65) | 0.7580 |
| Smoking, n (%) | 362 (46.06) | 130(55.56) | 0.012 | 16(1.2) | 11(4.25) | 0.0005 |
| Chronic diseases, n (%) | 320(40.71) | 104(44.4) | 0.5727 | 665 (49.89) | 127 (49.03) | 0.6517 |
| Poor sleep quality, n (%) | 190(24.17) | 82(35.04) | 0.0325 | 387(29.03) | 99(38.22) | 0.481 |
| Cognitive Impairment, n (%) | 31 (3.94) | 21(8.97) | 0.0021 | 157(11.78) | 46(17.76) | 0.0083 |
| ADL disability, n (%) | 44 (5.60) | 32(13.68) | <0.001 | 111(8.32) | 19(7.34) | 0.5940 |
| Social support status | 43.18±6.739 | 42.35±7.214 | 0.1180 | 43.41±7.027 | 40.9±7.701 | <0.001 |

**Table S3. Characteristics of the participants at baseline by sarcopenia severity** **subgroups.**

|  | Control  (N=2119) | Nonsevere sarcopenia  (N=240) | Severe sarcopenia  (N=253) | P |
| --- | --- | --- | --- | --- |
| Age, years | 60.68±7.55 | 65.08 ± 7.02 | 70.05 ± 8.14 | <0.001 |
| Male, n (%) | 786 (37.09) | 116 (48.33) | 118 (46.64) | <0.001 |
| SMMI, kg/m2 | 6.79±5.56 | 4.76 ±11.96 | 5.49 ± 2.02 | <0.0001 |
| Grip strength, kg | 24.29±8.84 | 24.8 ± 7.74 | 16.65 ± 5.45 | <0.001 |
| 4-m walking speed, m/s | 0.91±0.27 | 0.85 ± 0.22 | 0.72 ± 0.16 | <0.001 |
| Divorced, n (%) | 42 (1.98) | 0(0) | 2(0.79) | 0.039 |
| Widowed, n (%) | 258 (12.18) | 40 (16.67) | 48 (18.97) | 0.003 |
| Living alone, n (%) | 87 (4.11) | 9 (3.75) | 13 (5.14) | 0.6979 |
| Illiteracy, n (%) | 487 (22.99) | 51 (21.25) | 85 (33.6) | 0.001 |
| Farming, n (%) | 1270 (59.96) | 147 (61.25) | 181(71.54) | 0.002 |
| Drinking, n (%) | 427 (20.64) | 57(23.75) | 56 (22.13) | 0.4864 |
| Smoking, n (%) | 378 (17.85) | 71 (29.71) | 70 (27.67) | <0.001 |
| Chronic diseases, n (%) | 985(46.51) | 109(45.42) | 122(48.22) | 0.8150 |
| Poor sleep quality, n (%) | 577(27.24) | 68(28.33) | 73(28.85) | 0.236 |
| Cognitive Impairment, n (%) | 188 (8.87) | 22(9.17) | 45(17.79) | <0.001 |
| ADL disability, n (%) | 155 (7.31) | 19 (7.92) | 32 (12.65) | 0.012 |
| Social support status | 43.32 ± 6.92 | 42.99±7.17 | 40.26±7.58 | <0.0001 |

Abbreviations: Values are mean ± SD or valid percentages (n), BMI, body mass index; SMMI, skeletal muscle mass index; ADL, Activities of Daily Living.

**Table S4.** **Characteristics of the participants at baseline by muscle mass and muscle strength subgroups.**

|  | Control 1  (N=2037) | Low muscle mass (N=575) | P | Control 2  (N=1632) | Low muscle strength (N=980) | P |
| --- | --- | --- | --- | --- | --- | --- |
| Age, years | 60.75 ± 7.56 | 67.05 ± 7.07 | <0.001 | 60.2± 7.25 | 65.37 ± 8.36 | <0.001 |
| Male, n (%) | 744 (36.52) | 276 (48) | <0.001 | 642 (39.34) | 378 (38.57) | 0.364 |
| Divorced, n (%) | 337 (16.54) | 6 (1.04) | <0.001 | 226 (13.85) | 117 (11.94) | 0.135 |
| Widowed, n (%) | 40 (1.96) | 4 (0.7) | 0.037 | 29 (1.78) | 15 (1.53) | 0.754 |
| Living alone, n (%) | 252 (12.38) | 94 (16.35) | 0.013 | 178 (10.91) | 168 (17.16) | <0.001 |
| Illiteracy, n (%) | 84 (4.13) | 25 (4.35) | 0.814 | 64 (3.92) | 45(4.60) | 0.255 |
| Farming, n (%) | 476 (23.38) | 147 (25.57) | 0.278 | 341 (20.89) | 282 (28.8) | <0.001 |
| Drinking, n (%) | 413 (20.29) | 137 (23.83) | 0.067 | 380 (23.28) | 170 (17.36) | <0.001 |
| Smoking, n (%) | 355 (17.44) | 164 (28.57) | <0.001 | 334 (20.47) | 185 (18.90) | 0.176 |
| Chronic diseases, n (%) | 952 (46.76) | 264 (45.91) | 0.720 | 725 (44.42) | 491 (50.15) | 0.008 |
| Poor sleep quality, n (%) | 557(27.34) | 161(28.00) | 0.009 | 434(26.59) | 284(28.98) | 0.098 |
| Cognitive Impairment, n (%) | 184 (9.03) | 71 (12.35) | 0.018 | 125 (7.66) | 130 (13.27) | <0.001 |
| ADL disability, n (%) | 155(7.61) | 51(8.87) | 0.322 | 106 (6.5) | 100(10.2) | 0.001 |
| Social support status | 43.35 ± 6.90 | 41.73 ± 7.49 | <0.001 | 43.79 ± 6.92 | 41.68 ± 7.11 | <0.001 |

Abbreviations: Values are mean ± SD or valid percentages (n), BMI, body mass index; SMMI, skeletal muscle mass index; ADL, Activities of Daily Living.

**Table S5. Characteristics of the participants at baseline between follow-up and lost to follow-up.**

|  | With follow-up  (N=2612) | Lost to follow-up (N=751) | *P* |
| --- | --- | --- | --- |
| Age, years | 62.14±8.08 | 63.02±8.82 | 0.01 |
| Male, n (%) | 1012(38.74) | 261(34.75) | 0.018 |
| BMI, kg/m2 | 25.76±4.11 | 24.91±3.48 | ＜0.001 |
| Sarcopenia, n (%) | 493 (18.87) | 132 (17.58) | 0.089 |
| Divorced, n (%) | 44(1.68) | 11(1.46) | 0.41 |
| Widowed, n (%) | 346(13.25) | 88(11.72) | 0.148 |
| Living alone, n (%) | 104(3.98) | 26(3.46) | 0.297 |
| Illiteracy, n (%) | 623(23.85) | 191(25.43) | 0.201 |
| Drinking, n (%) | 550(21.06) | 176(23.44) | 0.091 |
| Smoking, n (%) | 519(19.87) | 146(19.44) | 0.416 |
| Chronic diseases, n (%) | 255(9.76) | 74(9.85) | 0.494 |
| Poor sleep quality, n (%) | 718(27.49) | 210(27.96) | 0.329 |
| Cognitive Impairment, n (%) | 206(7.89) | 71(9.45) | 0.098 |
| ADL disability, n (%) | 1216(46.55) | 292(38.88) | ＜0.001 |
| Social support status | 43.00±7.07 | 41.73±7.08 | ＜0.001 |

Abbreviations: Values are mean ± SD or valid percentages (n), BMI, body mass index; SMMI, skeletal muscle mass index; ADL, Activities of Daily Living.

**Table S6. Univariate analysis for** [**depressive symptom**](javascript:;)**.**

| Parameter | Univariable | | 95.0% CI | |
| --- | --- | --- | --- | --- |
|  | P | RR | Lower | Upper |
| Sarcopenia | 0.0823 | 1.228 | 0.974 | 1.548 |
| Age | 0.365 | 0.994 | 0.982 | 1.007 |
| Gender | 0.0035 | 1.367 | 1.11 | 1.687 |
| BMI | <0.001 | 1.532 | 1.202 | 1.952 |
| Divorced | <0.001 | 2.244 | 1.407 | 3.589 |
| Widowed | 0.003 | 1.460 | 1.141 | 1.867 |
| Living alone | 0.002 | 1.760 | 1.230 | 2.520 |
| Illiteracy | 0.028 | 1.271 | 1.027 | 1.574 |
| Farming | 0.923 | 0.990 | 0.811 | 1.209 |
| Drinking | 0.027 | 0.741 | 0.569 | 0.967 |
| Smoking | 0.317 | 0.881 | 0.683 | 1.137 |
| Chronic diseases | 0.007 | 1.308 | 1.076 | 1.590 |
| Sleep quality | <0.001 | 1.850 | 1.464 | 2.338 |
| Cognitive Impairment | <0.001 | 1.608 | 1.234 | 2.095 |
| ADL disability | 0.004 | 1.545 | 1.152 | 2.070 |
| Social support status | <0.001 | 0.973 | 0.960 | 0.986 |

Abbreviations: BMI, body mass index; SMMI, skeletal muscle mass index; ADL, Activities of Daily Living.
